# Case Report: Analytical Electron Microscopy of Lung Granulomas Associated with Exposure to Coating Materials Carried by Glass Wool Fibers

**DOI:** 10.1289/ehp.0901110

**Published:** 2009-10-13

**Authors:** Angela S. Ferreira, Valéria B. Moreira, Marcos César S. Castro, Porfírio J. Soares, Eduardo Algranti, Leonardo R. Andrade

**Affiliations:** 1 Departamento de Medicina Clínica and; 2 Departamento de Patologia, Hospital Universitário Antônio Pedro, Universidade Federal Fluminense, Niterói, Rio de Janeiro, Brasil; 3 Serviço de Medicina, Fundacentro/Centro Tecnico Nacional, São Paulo, Brasil; 4 Instituto de Ciências Biomédicas, Centro de Ciências da Saúde, Universidade Federal do Rio de Janeiro, Ilha do Fundão, Rio de Janeiro, Brasil

**Keywords:** analytical electron microscopy, coating materials, granuloma, man-made vitreous fibers, pneumoconiosis

## Abstract

**Context:**

Man-made vitreous fibers (MMVFs) are noncrystalline inorganic fibrous material used for thermal and acoustical insulation (e.g., rock wool, glass wool, glass microfibers, and refractory ceramic fibers). Neither epidemiologic studies of human exposure nor animal studies have shown a noticeable hazardous effect of glass wools on health. However, MMVFs have been anecdotally associated with granulomatous lung disease in several case reports.

**Case presentation:**

Here, we describe the case of a patient with multiple bilateral nodular opacities who was exposed to glass wool fibers and coating materials for 7 years. Bronchoalveolar lavage fluid revealed an increased total cell count (predominantly macrophages) with numerous cytoplasmic particles. Lung biopsy showed peribronchiolar infiltration of lymphoid cells and many foreign-body–type granulomas. Alveolar macrophages had numerous round and elongated platelike particles inside the cytoplasm. X-ray microanalysis of these particles detected mainly oxygen/aluminum/silicon and oxygen/magnesium/silicon, compatible with kaolinite and talc, respectively. No elemental evidence for glass fibers was found in lung biopsy.

**Discussion:**

The contribution of analytical electron microscopy applied in the lung biopsy was imperative to confirm the diagnosis of pneumoconiosis associated with a complex occupational exposure that included both MMVFs and coating materials.

**Relevance to clinical or professional practice:**

This case study points out the possible participation of other components (coating materials), beyond MMVFs, in the etiology of pneumoconiosis.

Man-made vitreous fibers (MMVFs), also called man-made mineral fibers, represent a group of manufactured fibers that includes rock wool, slag wool, glass wool, glass continuous filaments, glass microfibers, and refractory ceramic fibers (Baan and Grosse 2004; De Vuyst et al. 1995). The uses of MMVFs are mainly related to thermal and acoustic insulation (Hesterberg and Hart 2001). Because past inhalation of asbestos could be associated with lung diseases, concerns have been raised about possible deleterious effects in the respiratory system associated with MMVFs (Moore et al. 2002). The International Agency for Research on Cancer (IARC 2002) classified slag and rock wools as Group 3 (unclassifiable as human carcinogens) and ceramic fibers as Group 2B (possible human carcinogens, limited evidence) based on animal model studies. Carel et al. (2007) conducted a multicenter study in Europe to elucidate the extent to which lung cancer burden in men was driven by asbestos and MMVFs, but they did not observe a significant overall increase in risk of lung cancer caused by MMVFs. MMVFs were associated with granulomatous lung disease in humans in some reported cases (Drent et al. 2000a, 2000b; Guber et al. 2006; Klimczak et al. 2000; Takahashi et al. 1996; Vahid et al. 2007). In an experimental study, Adamson et al. (1995) administered a milled fiberglass sample to mice by intratracheal instillation and observed granulomas at bronchoalveolar ducts and morphologic evidence of fibrosis.

In the present study, we describe a case of diffuse pulmonary nodular lesions caused by exposure to MMVF and coating materials applied in the speedboat industry. A number of coating materials are used in the production of glass-wool fibers and also in paints and varnishes used in the manufacture of boats. Energy-dispersive X-ray analysis (EDXA) associated with transmission electron microscopy (TEM) and scanning electron microscopy (SEM) performed on original fibers, coating materials, and lung biopsy was fundamental to identifying the elemental composition of these materials and diagnosing this pneumoconiosis.

## Case Report

A 36-year-old man was admitted to Antonio Pedro University Hospital (Rio de Janeiro, Brazil) in November 2004 with bilateral diffuse pulmonary infiltrates on the chest radiograph and dyspnea on exertion. He had been working as a laminator of glass-wool fibers and coating materials for 7 years, without respiratory protection, suggesting excessive occupational exposures to MMVFs and coating materials. His past medical history was unremarkable, and he was a lifetime nonsmoker.

**Figure 1:**
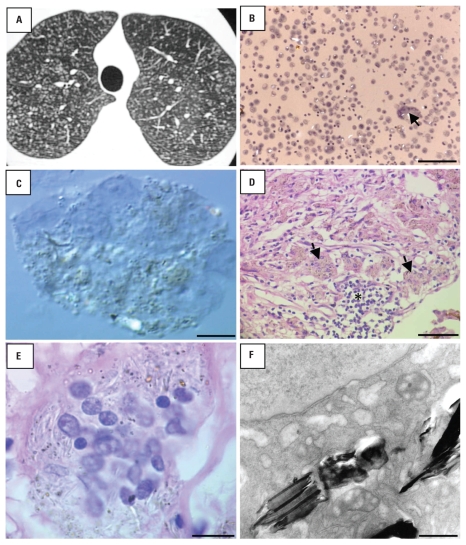
Images resulting from various tests performed on the patient. (*A*) HRCT image showing small nodular opacities throughout lung fields. (*B*) Bright-field microscopy image of BALF showing numerous macrophages and a multinucleated giant cell (arrow) containing bright refractile particles; bar = 100 μm. (*C*) Differential interferential contrast microscopy of a multinucleated giant cell in BALF with refractile particles within the cytoplasm; bar = 5 μm. (*D*) Light microscopy of the lung biopsy specimen (hematoxylin and eosin stain) showing many foreign-body–type granulomas (asterisk) and giant cells (black arrows); bar = 100 μm. (*E*) Detail of a multinucleated giant cell with numerous round and elongated particles within the cytoplasm (hematoxylin and eosin stain); bar = 7.5 μm. (*F*) TEM image of a macrophage showing platelike materials within the cytoplasm; bar = 0.25 μm.

On admission, he was in good clinical condition, and his physical examination was normal. Chest radiograph and high-resolution computed tomography (HRCT) showed small nodular opacities spread throughout the lung fields (Figure 1A). Routine laboratory data showed normal hematologic, hepatic, and renal function. Results of sputum analyses for acid-fast organisms and neoplasia were negative. Pulmonary function studies were normal.

The patient underwent bronchoalveolar lavage, and the sample was used for cell count, cytologic examination, and specific staining for fungi and acid-fast bacilli. Cellular bronchoalveolar lavage fluid (BALF) observed by light microscopy revealed an increased total cell count, predominantly macrophages (differential cell count: macrophages, 90%; lymphocytes, 9%; neutrophils, 1%; eosinophils, 0%). Numerous particles of different sizes were observed within the cytoplasm of alveolar macrophages by differential interferential contrast microscopy (Figure 1C). Some of these particles were anisotropic. Stains were negative for fungi and acid-fast bacilli.

An open lung biopsy was performed because of an uncommon radiologic pattern rarely observed in patients with an occupational history of glass fiber exposure. Light microscopy showed peribronchiolar infiltration of lymphoid cells and many foreign-body–type granulomas throughout the examined tissue (Figure 1D). Alveolar macrophages observed by light microscopy (Figure 1E) had numerous round and elongated particles inside their cytoplasm, and platelike material when examined by TEM (length, > 1 to 12 μm) (Figure 1F).

**Figure 2:**
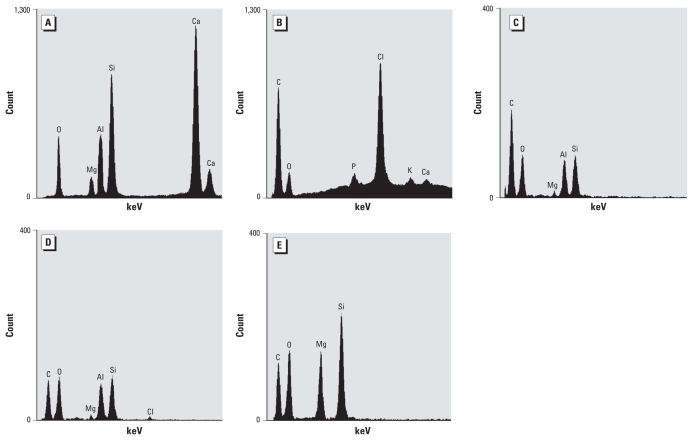
EDXA spectra obtained by TEM. (*A*) EDXA spectrum obtained from the fibers brought in by the patient. (*B*) Spectrum from the resin also used in the lamination process. (*C*) Spectrum from a platelike material in the patient’s alveolar macrophages derived from the lung biopsy; this spectrum is compatible with kaolinite. (*D*) Spectrum of a different platelike material in the lung alveolar macrophage, suggesting the presence of resin because of Cl. (*E*) Spectrum from an amorphous material in the lung alveolar macrophages indicating a talc-like material.

Glass wool fiber and the resin used in lamination were brought in by the patient and studied. These materials were deposited on a carbon (C)-conductive tape covering SEM stubs and analyzed by EDXA (Noran-Voyager analytical system; Thermo Scientific, Waltham, MA, USA) coupled in a Jeol 1200 EX scanning-transmission electron microscope (Jeol, Tokyo, Japan). The fibers contained the elements oxygen (O), magnesium (Mg), aluminum (Al), silicon (Si), and calcium (Ca), matching with glass wool fibers (Figure 2A). The resin had C, O, phosphorus (P), potassium (K), Ca, and high chlorine (Cl) X-ray counts (Figure 2B). The analyses performed on the platelike material within macrophages detected C, O, Mg, Al, and Si, compatible with the mineral kaolinite (Figure 2C), and also Cl (Figure 2D), suggesting a resin residue. Some amorphous materials in the macrophages contained C, O, Mg, and Si, indicating a talc-like material (Figure 2E). Analysis performed in an empty area of the cytoplasm detected C and O only (data not shown). Four years after the patient stopped working with fiberglass, his overall condition was the same, and a follow-up computed tomographic (CT) scan of the chest showed the same initial pattern (data not shown).

## Discussion

Epidemiologic studies in humans suggest that there is no direct evidence of chronic lung disease associated with glass exposure (Lippmann 1990; Marsh et al. 2001; Morgan et al. 1981). A cohort study of 6,586 workers engaged in glass fiber production indicated no excess malignant or nonmalignant respiratory disease (Morgan et al. 1981). In several studies, chest roentgenograms of MMVF-exposed workers were evaluated. No evidence of an association between exposure and lesion was found (Hughes et al. 1993; Weill et al. 1983). Our patient presented multiple bilateral nodular opacities seen by chest radiograph and confirmed by HRCT. A lung biopsy was suggested in this patient because, as pointed out in a number of previous reports, workers in glass fiber plants had no demonstrable clinical and roentgenologic pulmonary manifestations (e.g., Carel et al 2007). Here, a granulomatous lung disease was described, and many foreign-body–type granulomas were found throughout the lung specimens. There are some reports regarding the possibility of developing granulomatous lung disease after MMVF exposure. For example, Takahashi et al. (1996) reported the case of a 56-year-old man who was a carpenter with long-term exposure to fiberglass. His chest radiograph showed small nodular opacities in lower lung fields. A transbronchial lung biopsy revealed interstitial fibrosis, but no granuloma was found. Drent et al. (2000b) described a case of a 31-year-old man exposed for 6 months, 11 years earlier, to small respirable fiberglass particles. An HRCT showed small nodular opacities, most in the middle and upper lung zone. Lung biopsy showed granulomas with multinucleated giant cells. Additional qualitative X-ray analysis of glass fibers within the lung revealed Si, Al, and titanium. A distinct relation between fiber deposits and granulomas was found. According to the authors, this observation strongly suggests that the presence of particles was not merely accidental, but was most probably associated with the development of the granulomas.

Klimczak et al. (2000) reported the case of a 39-year-old man with granulomatous lesions in both lungs; he had worked for 18 years with glass fibers. On chest CT, disseminated small nodular lesions were found. In the lung biopsy, many foreign-body–type granulomas were found throughout the sample. The authors also considered the possibility of development of such lesions after the exposure to glass fibers.

To determine the possible association of MMVF exposure and the development of sarcoid-like granulomas, Drent et al. (2000a) reviewed the records of 50 patients with sarcoidosis who visited their outpatient clinic between 1996 and 1999. Fourteen cases recalled a history of exposure to glass fibers or rock wool, both of which are MMVFs. In all lung biopsy sections, the main component was a nonconfluent nonnecrotizing granulomatous inflammation located at the submucosal interstitium and sometimes subpleurally. In some granulomas, Langhans-type or foreign-body–type multinucleated giant cells were present.

Guber et al. (2006) reported a case of interstitial lung disease with a relatively benign course during the follow-up period of ≥ 4 years. Because of the high percentage of CD8^+^ T lymphocytes in the induced sputum and BALF, the histologic findings and lung CT–scan changes together indicated a possible active inflammatory process, resembling pulmonary fibrosis. The fibers found in the biopsy slides of the patient resembled the morphology and chemical composition of those found in typical glass wool insulation materials. The authors concluded that the disease was probably caused by low-fibrogenic-activity glass wool fibers.

Vahid et al. (2007) described a case of noninfectious, noncaseating granulomatous lymphadenitis with giant cell formation and pulmonary disease (that mimicked the characteristics of sarcoidosis) in a patient with fiberglass exposure. According to these authors, the presence of fiberglass in lymph node tissue and resolution of the disease process after cessation of exposure supports the association of this sarcoidosis-like disease and fiberglass exposure.

In our study, beyond the inhalation of the small glass fibers, the patient also inhaled other materials used in the lamination process, which should be evaluated as possible confounders. EDXA spectra of lung tissue did not indicate exactly the same elements present in original glass fibers. Ca was not detected within the alveolar macrophages, indicating that Ca (and the fibers per se) could be dissolved by lysosomal acid hydrolases. Spectra showed mainly O/Al/Si in platelike fibrillar particles inside alveolar macrophages, compatible with kaolinite, an insoluble nonfibrous silicate. Mg was also detected in the original fibers and in alveolar macrophage particles but, combined with O and Si, should be part of a talc-like material aspirated by the patient, such as kaolinite, which is commonly used as fillers in paints and plastics in boat industry. Several radiologic studies have shown small rounded opacities in kaolin and talc pneumoconiosis, and a number of different lesions have been described in the lungs of persons exposed to talc (Gibbs et al. 1992). The lesions include macules, nodules, diffuse interstitial fibrosis, and progressive massive fibrosis. Microscopically, those lesions have shown perivascular and peribronchiolar collections of mineral-containing macrophages, accompanied by variable degrees of fibrosis, foreign body granulomas, mixed dust fibrotic lesions, and ferruginous bodies. Foreign body granulomas containing large numbers of birefringent crystalline particles have been described, but rarely sarcoid-like granulomas (Gibbs et al. 1992).

Inhalation of metal dust or fumes can cause granulomatous lung disease that mimics sarcoidosis. Particular metals that possess antigenic properties, which promote granuloma formation, include Al, barium, beryllium, cobalt, copper, gold, titanium, and zirconium (Newman 1998). Al was detected in the alveolar macrophages associated with Si, suggesting the presence of aluminosilicate particles. Kelleher et al. (2000) reported that Al-rich particles may induce noncaseating granulomas principally in workers involved in production and manufacturing of Al. However, few cases of pulmonary sarcoid-like granulomatosis induced by Al dust have been reported in the literature (Cai et al. 2007).

The biopersistence mechanism of the fibers deposited in the respiratory tract results from a combination of physiologic clearance (mechanical translocation/removal) and physicochemical events (chemical dissolution and leaching, mechanical breaking) (Baan and Grosse 2004).

We found fibers < 12 μm inside the alveolar macrophages. Human macrophages can phagocytose fibers ≤ 20 mm (Zeidler-Erdely et al. 2006). The potential pathogenicity of glass fibers is dependent on their length, clearance, solubility, and biopersistence. For fibers < 15 μm, the clearance performed by alveolar macrophages is the major primary mechanism, whereas for fibers > 20 μm, the mechanism is dissolution and fragmentation (reviewed by Bernstein 2007). The pH of the lung fluid is around 7.4, whereas the phagolysosomes and surfaces of activated macrophages are pH 4.5 and 5, respectively (Bernstein 2007). Dissolution of MMVFs in the lung environment, well demonstrated in animal studies (Adamson et al. 1995), is one of the explanations proposed to support the lack of adverse health effects of these fibers in epidemiologic studies. The lack of biopersistence of MMVFs also explains their rarity in the lung samples analyzed for asbestos and other fibers (Dumortier et al. 2001; McDonald and Grosse 1990).

According to De Vuyst et al. (1995), only three studies address quantification and characterization of MMVFs by analytical electron microscopy in lung samples of exposed workers. The largest of these studies (McDonald et al. 1990) analyzed fiber content in the lungs from 131 deceased MMVF workers (glass wool, rock wool, and slag wool) from a large U.S. cohort and from 112 matched referents. The authors of that study described the absence of MMVFs in the lungs of most exposed workers and concluded that the workers were exposed to nonrespirable fibers or that the inhaled fibers did not survive in the pulmonary environment.

## Conclusions

Based on our findings and on the few studies available in the literature, the use of X-ray microanalysis provides an accurate identification of the deposits observed within the alveolar macrophages and supports a causal association with the type of exposure reported by the patient and his lung disease. In our case, the contribution of analytical electron microscopy applied in lung biopsy confirmed the diagnosis of this pneumoconiosis associated with a complex occupational exposure, but not clearly due to MMVFs.
