# Errata

**Published:** 2010-10

In the article by Ferreira et al. [ Environ Health Perspect 118:249–252 (2010)], the units for fiber length were incorrect in the first column of page 252; “millimeters” should have been “micrometers.” The corrected sentence is as follows:

Human macrophages can phagocytose fibers ≤ 20 μm (Zeidler-Erdely et al. 2006).

The authors apologize for the error.

